# The Effect of Mesoporous Bioactive Glass Nanoparticles/Graphene Oxide Composites on the Differentiation and Mineralization of Human Dental Pulp Stem Cells

**DOI:** 10.3390/nano10040620

**Published:** 2020-03-27

**Authors:** Jae Hwa Ahn, In-Ryoung Kim, Yeon Kim, Dong-Hyun Kim, Soo-Byung Park, Bong-Soo Park, Moon-Kyoung Bae, Yong-Il Kim

**Affiliations:** 1 Department of Orthodontics, Dental Research Institute, Pusan National University, Yangsan 50612, Korea; anzzshock@naver.com (J.H.A.); sbypark@pusan.ac.kr (S.-B.P.); 2 Department of Oral Anantomy, School of Dentistry, Pusan National University, Yangsan 50612, Korea; biowool@pusan.ac.kr (I.-R.K.); parkbs@pusan.ac.kr (B.-S.P.); 3 Department of Oral Physiology, School of Dentistry, Pusan National University, Yangsan 50612, Korea; graceyeon88@gmail.com; 4 R&D Center, DAEWON MATERIALS Co., Ltd., 365, Sinseon-ro, Nam-gu, Busan 48547, Korea; kimnano75@gmail.com; 5 Dental and Life Science Institute, Pusan National University, Yangsan 50612, Korea

**Keywords:** bioactive glass, graphene oxide, hDPSC, odontogenic differentiation, mineralization

## Abstract

The purpose of this study was to investigate the effects of mesoporous bioactive glass nanoparticle (MBN)/graphene oxide (GO) composites on the mineralization ability and differentiation potential of human dental pulp stem cells (hDPSCs). MBN/GO composites were synthesized using the sol-gel method and colloidal processing to enhance the bioactivity and mechanical properties of MBN. Characterization using FESEM, XRD, FTIR, and Raman spectrometry showed that the composites were successfully synthesized. hDPSCs were then cultured directly on the MBN/GO (40:1 and 20:1) composites in vitro. MBN/GO promoted the proliferation and alkaline phosphatase (ALP) activity of hDPSCs. In addition, qRT-PCR showed that MBN/GO regulated the mRNA levels of odontogenic markers (dentin sialophosphoprotein (DSPP), dentine matrix protein 1 (DMP-1), ALP, matrix extracellular phosphoglycoprotein (MEPE), bone morphogenetic protein 2 (BMP-2), and runt-related transcription factor 2 (RUNX-2)). The mRNA levels of DSPP and DMP-1, two odontogenesis-specific markers, were considerably upregulated in hDPSCs in response to growth on the MBN/GO composites. Western blot analysis revealed similar results. Alizarin red S staining was subsequently performed to further investigate MBN/GO-induced mineralization of hDPSCs. It was revealed that MBN/GO composites promote odontogenic differentiation via the Wnt/β-catenin signaling pathway. Collectively, the results of the present study suggest that MBN/GO composites may promote the differentiation of hDPSCs into odontoblast-like cells, and potentially induce dentin formation.

## 1. Introduction

Stem cell-based dentin regeneration may provide an alternative to the conventional conservative treatment of teeth in cases of trauma or caries [1]. Human dental pulp stem cells (hDPSCs) secrete a dentin matrix and differentiate into odontoblasts through the stimulation of specific cytokines or growth factors [2]. Gronthos et al. [3] transplanted hDPSCs into the subcutaneous cavity of immunocompromised mice and observed the formation of dentin-pulp-like complexes. Since this initial observation, dentin regeneration using hDPSCs has been actively investigated in the field of dentin-pulp tissue engineering.

In recent decades, bioactive glass (BAG), consisting of 24.5% CaO, 24.5% Na_2_O, 6.0% P_2_O_5_, and 45% SiO_2_ has been widely studied as a biomaterial in tissue engineering, due to its resorbable properties and ion-release capability [4]. As a result of its ion-release capability, BAG can form a bioactive hydroxyapatite layer, and experimental results have shown its effect on the remineralization of dentin and enamel [5]. BAG has recently been shown to induce stem cell differentiation, suggesting that it may be used for the repair and regeneration of bone tissue [6]. However, BAG does not have sufficient mechanical strength to replace bone or tissue and is therefore inadequate for weight-loaded environments [7]. To overcome this limitation, several polymers and inorganic materials have been introduced as reinforcements to increase the mechanical strength of BAG [8]. Currently, graphene and its derivatives are some of the thinnest and mechanically strongest materials; they are receiving increasing attention as reinforcing agents. These materials have been shown to be superior in terms of transferring mechanical strength to host materials compared with other reinforcing agents [9]. In particular, graphene and its derivatives have excellent biocompatibility and biostability, and have been shown to enhance cell attachment, proliferation, and differentiation in various stem cell studies. Additionally, their effects on osteogenic differentiation have been reported in a number of previous studies [9,10]. 

Several studies have reported that various graphene oxide (GO)-reinforced composites—synthesized to enhance mechanical strength—induce the osteogenic differentiation of mesenchymal stem cells [11]. However, few reports have investigated the effect of these composites on odontogenic differentiation in the same cell type.

The purpose of the present study was to investigate the cellular response of hDPSCs cultured on mesoporous bioactive glass nanoparticle (MBN)/GO composites and their effect on odontogenesis. The specific aims of the present study were (1) to synthesize MBN/GO composites using GO as a reinforcement to enhance the biological and mechanical properties of MBN and (2) to evaluate the effect of these composites on the mineralization ability and differentiation potential of hDPSCs.

## 2. Materials and Methods 

### 2.1. Synthesis of MBN

MBN was synthesized using a modified sol-gel process. Briefly, 3.12 g calcium nitrate tetrahydrate (Ca(NO_3_)_2_·4H_2_O) (Sigma-Aldrich, St. Louis, MO, USA), 2 mL aqueous ammonia (Samchun, Seoul, Korea), 10 mL 2-ethoxyethanol (Sigma-Aldrich, St. Louis, MO, USA), 20 mL ethanol (Samchun, Seoul, Korea), and 1 g hexadecyltrimethylammonium bromide (CTAB) (Sigma-Aldrich, St. Louis, MO, USA) were mixed in 150 mL distilled water. The mixture was stirred at room temperature for 30 min. Then, 5 mL tetraethyl orthosilicate (TEOS; Sigma-Aldrich, St. Louis, MO, USA) was added and stirred at room temperature for 30 min. Subsequently, 0.25 mL triethyl phosphate (TEP; Sigma-Aldrich, St. Louis, MO, USA) was added and the mixture was vigorously stirred for 4 h at room temperature. A white precipitate was formed and dried in a vacuum oven at 60 °C for 24 h. The dried gel powder was calcined at 600 °C for 5 h. The molar ratio of SiO_2_:CaO:P_2_O_4_ was calculated to be 60:36:4.

### 2.2. Preparation and Characterization of the MBN/GO Composite

The MBN/GO composite powder was synthesized using the colloidal processing method. MBN, synthesized using the aforementioned method, and commercially purchased GO suspension (Graphenea, San Sebastián, Spain) were used as the starting materials. MBN suspension (60 and 80 mg/mL) and GO suspension (2 mg/mL) were individually sonicated for 2 h using deionized (DI) water as a solvent. After sonication, the GO suspension was added dropwise to the MBN suspension using a separatory funnel, with the weight ratio of the MBN/GO composite of 40:1 and 20:1, respectively, depending on the concentration of MBN. This process was performed slowly under magnetic stirring, and the mixture was dried in a vacuum oven at 65 °C for 24 h. The resulting powder was ground and filtered to obtain the MBN/GO composite powder with a weight ratio of 40:1 and 20:1.

The surface morphology of the MBN/GO composites was observed using a field emission scanning electron microscope (FESEM) (SU-70; Hitachi, Tokyo, Japan). X-ray diffraction spectroscopy (XRD) (X’Pert PRO MPD; Panalytical, Malvern, UK) with CuKα radiation in the range of 2θ = 10–45° at 40 kV and 40 mA, attenuated total reflectance-Fourier transformation infrared spectroscopy (ATR-FTIR) (FT/IR-6300; JASCO, Tokyo, Japan), and Raman spectroscopy (NRS-3300; JASCO, Tokyo, Japan) were used to analyze and characterize the sample.

### 2.3. MBN/GO Composite Coating

After sterilization at high temperature, the MBN and MBN/GO powder suspensions were suspended in DI water, both at final concentrations of 0.01 and 0.05% (w/v). For surface coating at the same concentrations, 100, 588, and 2972 μL of the suspensions were added to 96-, 24-, and 6-well polystyrene culture plates (Thermo Fisher Scientific, Waltham, MA, USA), respectively (Table 1). The culture plates were dried in a vacuum oven at 65 °C for 24 h, until a uniform adherent layer of each composite was obtained.

### 2.4. Cell Culture

hDPSCs were cultured in Dulbecco’s modified Eagle’s medium (DMEM, Gibco, Grand Island, NY, USA) supplemented with 10% fetal bovine serum (FBS, Gibco, Grand Island, NY, USA) and 1% Penicillin-Streptomycin (Gibco, Grand Island, NY, USA) and maintained in an incubator at 37 °C and 5% CO_2_. hDPSCs were passaged every 2–5 days and cells at passages 5–7 were used for subsequent experiments.

### 2.5. Cell Viability Assay

The 3-(4–dimethylthiazol-2-yl)-2,5-diphenyl tetrazolium bromide (MTT) colorimetric assay was performed to assess cell viability. hDPSCs were seeded at a density of 1 × 10^4^/well in a 96-well culture plate coated with MBN and MBN/GO powder. After 24 and 48 h, the culture medium in each well was replaced with 100 μL fresh culture medium containing 10 μL MTT solution (5 mg/mL MTT in sterile Phosphate-buffered saline (PBS), Sigma-Aldrich, St. Louis, MO, USA) and incubated for 4 h at 37 °C and 5% CO_2_. Then, the medium was replaced with 100 μL dimethyl sulfoxide (Sigma-Aldrich, St. Louis, MO, USA) to dissolve the formazan crystals for 5 min at 37 °C. The absorbance was subsequently measured at a wavelength of 620 nm using a microplate reader (Sunrise; TECAN, Männedorf, Switzerland). Three independent experiments were performed.

### 2.6. Alkaline Phosphatase (ALP) Activity Assay

hDPSCs were seeded at a density of 5 × 10^4^ cells/well in a 24-well plate coated with MBN and MBN/GO powder. hDPSCs were cultured in alpha-Minimum Essential Medium (α-MEM, Gibco, Grand Island, NY, USA) containing 10 mM β-glycerophosphate, 50 μg/mL ascorbic acid, and 0.1 μM dexamethasone to induce differentiation. The medium was changed every two days. The odontogenic differentiation of hDPSCs was assessed after 7 and 14 days by measuring ALP activity using an ALP Detection Kit (Sigma-Aldrich, St. Louis, MO, USA) according to the manufacturer’s protocol. Briefly, 50 μL ALP substrate solution (1 vial (24 mg) p-nitro-phenyl phosphate (pNPP) dissolved in 5 mL ALP buffer containing 0.2 M Tris-HCl, pH 9.5, 1 mM MgCl_2_) were added to 40 μL of cell lysate obtained using an extraction solution and incubated at 37 °C for 60 min. Then, 50 μL stop solution (0.5 N NaOH) were added to each well to stop the reaction, and the absorbance of the supernatant was measured at a wavelength of 405 nm. ALP activity was calculated from the standard calibration curve.

### 2.7. Quantitative Real-Time Polymerase Chain Reaction (qRT-PCR)

hDPSCs were seeded at a density of 2 × 10^5^ cells/well in 6-well plates coated with MBN and MBN/GO powder and cultured in induction medium for 7 and 14 days, followed by total RNA extraction using a RNeasy Mini kit (Qiagen, Hilden, Germany). A total of 1 µg isolated RNA was reverse transcribed into cDNA using the M-MLV cDNA Synthesis kit (Enzynomics, Daejeon, Korea), according to the manufacturer’s instructions. qRT-PCR was subsequently performed using the Power SYBR Green kit (Applied Biosystems, Foster City, CA, USA) on a 7500 Real-Time PCR system (Applied Biosystems). The relative expression of dentin sialophosphoprotein (DSPP), dentine matrix protein 1 (DMP-1), ALP, matrix extracellular phosphoglycoprotein (MEPE), bone morphogenetic protein 2 (BMP-2), and runt-related transcription factor 2 (RUNX-2) was determined using β-actin as the internal reference gene. The primers used for qRT-PCR are presented in Table 2. All experiments were repeated in triplicate.

### 2.8. Western Blot

hDPSCs were seeded at a density of 2 × 10^5^ cells/well in a 6-well plate coated with MBN and MBN/GO powder and cultured for 7 and 14 days. The cells were subsequently harvested, washed with PBS, and lysed using radio immunoprecipitation assay (RIPA) lysis buffer (Thermo Fisher Scientific, Waltham, MA, USA) containing protease inhibitors. Protein concentration was quantified using a Bio-Rad protein assay kit (Thermo Fisher Scientific, Waltham, MA, USA). Briefly, protein (15 µg/lane) was loaded onto 10% SDS-PAGE gels at 120 V for 90 min and the separated proteins were transferred onto Polyvinylidene difluoride (PVDF) membranes (Millipore, Bedford, MA, USA) at 80 V for 90 min. The membranes were blocked in blocking buffer (5% w/v skim milk, 0.1% Tween-20 in PBS pH 7.4) at room temperature for 1 h and incubated with the following primary polyclonal antibodies: anti-DMP-1 (1:5000, ab103203, Abcam, Cambridge, MA, USA), anti-DSPP (1:1000, sc73632, Santa Cruz Biotechnology Inc., Dallas, TX, USA), and anti- Glyceraldehyde 3-phosphate dehydrogenase (GAPDH) (1:2000, Santa Cruz Biotechnology Inc., Dallas, TX, USA) overnight at 4 °C. The membranes were washed with PBST (PBS containing 0.2% Tween-20) five times for 1 h. Then, the membranes were incubated with the appropriate horseradish peroxidase (HRP)-conjugated secondary antibodies (Santa Cruz Biotechnology Inc., Dallas, TX, USA). The membranes were washed with PBST and the protein bands were visualized using SuperSignal West Femto substrate (Pierce, Rockford, IL, USA) and captured using an ImageQuant LAS 500 imager system (GE Healthcare, Little Chalfont, UK).

### 2.9. Alizarin Red S Staining and Quantitative Analysis

hDPSCs were seeded at a density of 5 × 10^4^ cells/well in a 24-well plate coated with MBN and MBN/GO powder and cultured in induction medium, α-MEM containing 10 mM β-glycerophosphate and 50 μg/mL ascorbic acid. The medium was changed every two days. After 7 and 14 days of culture, the cells were washed twice with PBS and fixed with 4% paraformaldehyde for 15 min. The plates were then rinsed three times with DI water, followed by the addition of 500 µL 1% Alizarin red S to each well. The samples were stained for 10 min at room temperature before washing three times with DI water. After drying the samples, the culture plate was imaged. For quantification, 250 µL 10% acetic acid solution was added to each well, and the absorbance was measured at a wavelength of 492 nm using a microplate reader (Sunrise; Tecan, Männedorf, Switzerland). 

### 2.10. Analysis of Wnt/β-Catenin Signaling Pathway-Related GENE expression in hDPSCs Cultured on MBN/GO Composites

To investigate the effect of the MBN/GO composite on the expression of Wnt/β-catenin signaling pathway-related genes in hDPSCs, the expression of the Wnt/β-catenin-related genes, AXIN-2 and β-catenin, was determined using qRT-PCR as aforementioned. The primers used are presented in Table 2.

### 2.11. Statistical Analysis

Each experiment was performed in triplicate. Differences among the groups were compared using one-way analysis of variance (ANOVA) followed by Duncan’s new multiple range test. A *p*-value <0.05 was considered to indicate a statistically significant difference. All statistical analyses were performed using R (version 3.5.1; R Foundation for Statistical Computing, Vienna, Austria).

## 3. Results

### 3.1. Characterization of the MBN/GO Composite

#### 3.1.1. FESEM

**Figure 1:**
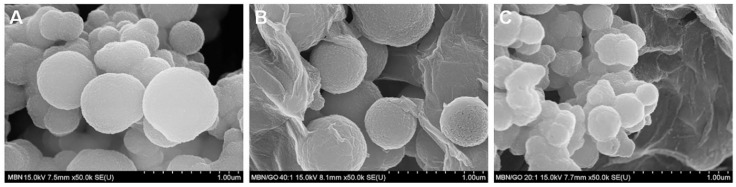
Field-emission scanning electron microscopy (FESEM) images of (**A**) MBN, (**B**) MBN/GO 40:1, and (**C**) MBN/GO 20:1.

The morphology of the MBN and MBN/GO composites was assessed using FESEM. The diameter of MBNs was in the range of 300–600 nm. FESEM revealed that spherical MBNs were coated by a planar sheet of GO (Figure 1).

#### 3.1.2. XRD

**Figure 2:**
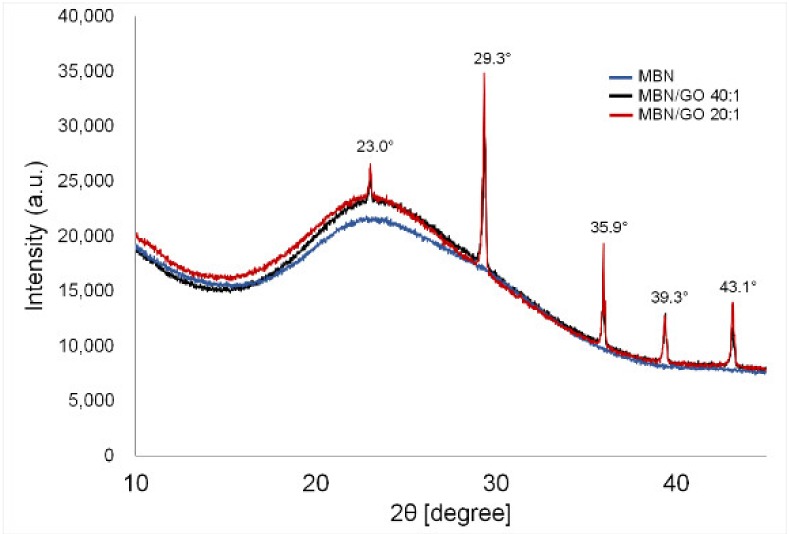
XRD spectra of MBN, MBN/GO 40:1, and MBN/GO 20:1.

The XRD patterns of the MBN and MBN/GO composites are shown in Figure 2. No diffraction peaks were observed in the graph, suggesting that MBNs are amorphous in form, similar to glass [12]. Both MBN/GO composites were amorphous, similar to MBNs alone. Calcite peaks of the crystalline phase (CaCO_3_, JCPDS #05-0586) were observed at 23.0, 29.3, 35.9, 39.3, and 43.1° [13,14,15,16].

#### 3.1.3. FTIR

**Figure 3:**
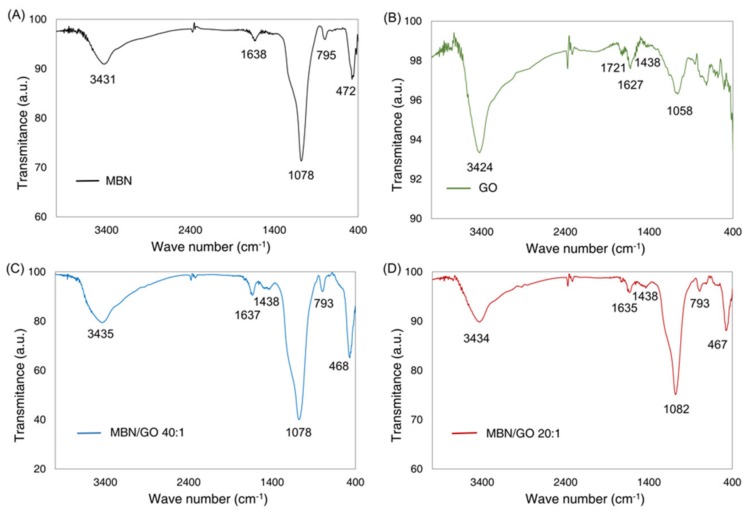
FTIR spectra of (**A**) MBN, (**B**) GO, (**C**) MBN/GO 40:1, and (**D**) MBN/GO 20:1.

The chemical structures of the MBN and MBN/GO composites were revealed by FTIR spectroscopy (Figure 3). The 3430 cm^−1^ peaks found in the FTIR spectra of MBN and GO resulted from the O–H stretching vibration of adsorbed water molecules. In the FTIR spectra of MBN, a very strong absorption peak was observed at 1078 cm^−1^, which was caused by Si–O–Si asymmetrical stretching vibrations [17]. After the incorporation of GO into the MBN, the conjugated carbonyl group of GO (corresponding to 1721 cm^−1^) disappeared and a new peak appeared at around 1080 cm^−1^, corresponding to the Si–O–Si asymmetrical stretching vibrations of MBN, indicating that this stretching vibration of the GO carbonyl groups was converted into Si–OC bands [18]. Moreover, the characteristic peaks of GO at 1058 and 1627 cm^−1^ disappeared after the formation of MBN/GO composites [19,20].

#### 3.1.4. Raman Spectroscopy

**Figure 4:**
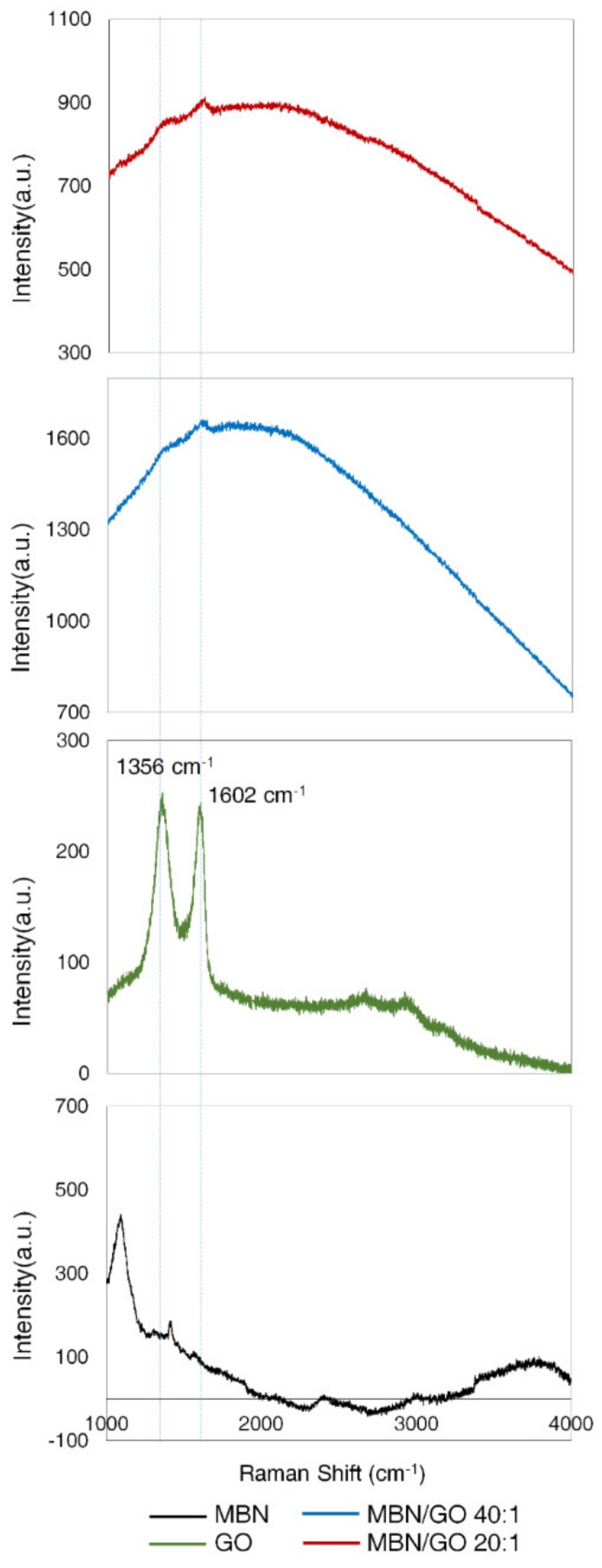
RAMAN spectra of MBN, GO, MBN/GO 40:1, and MBN/GO 20:1.

The characteristic peaks of GO were detected at 1356 cm^−1^ and 1602 cm^−1^, corresponding to the D and G bands, respectively [20]. The characteristic peaks of GO did not shift after MBN/GO composite formation, demonstrating that MBN and GO successfully formed composites (Figure 4).

### 3.2. Viability of hDPSCs

**Figure 5:**
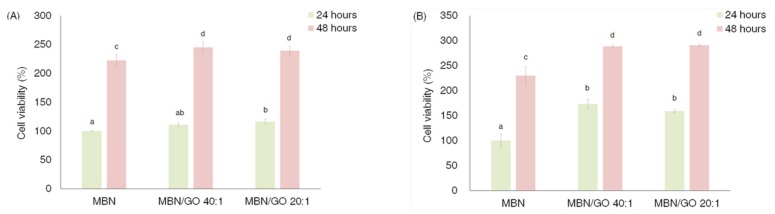
Cell viability analysis. Human dental pulp stem cells (hDPSCs) were cultured on different concentrations of MBN/GO after 24 h and 48 h: MBN, MBN/GO 40:1, and MBN/GO 20:1. (**A**) 0.1 mg/mL concentration of MBN, MBN/GO 40:1, and MBN/GO 20:1. (**B**) 0.5 mg/mL concentration of MBN, MBN/GO 40:1, and MBN/GO 20:1. ANOVA was performed to evaluate cell biocompatibility of different concentrations of MBN, MBN/GO 40:1, and MBN/GO 20:1 and test the statistical significance of samples between 24 h and 48 h. The same letters indicate that the *p*-value is not significantly different (*p* < 0.05). Error bars represent the standard deviation.

The viability of hDPSCs cultured on the MBN and MBN/GO composites at 24 and 48 h is presented in Figure 5. Both concentrations (0.1 and 0.5 mg/mL) resulted in the same pattern of enhanced cell viability over time, and the viability of cells grown on both MBN/GO groups was significantly more enhanced than that observed in the case of cells grown on MBNs alone, except for the MBN/GO 40:1 composite at 0.1 mg/mL at 24 h. However, the GO content did not significantly affect cell viability (Figure 5).

### 3.3. ALP Activity in hDPSCs

**Figure 6:**
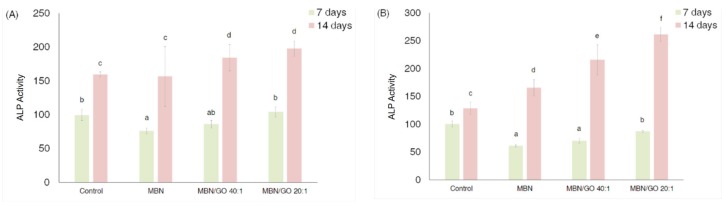
The ALP activity of hDPSCs cultured on polystyrene tissue culture plates (blank control), MBN, MBN/GO 40:1, and MBN/GO 20:1 at 7 and 14 days. (**A**) 0.1 mg/mL concentration of MBN, MBN/GO 40:1, and MBN/GO 20:1. (**B**) 0.5 mg/mL concentration MBN, MBN/GO 40:1, and MBN/GO 20:1. ANOVA was performed to evaluate the ALP activity of different concentrations of MBN, MBN/GO 40:1, and MBN/GO 20:1 and test the statistical significance of ALP activity between 24 h and 48 h. The same letters indicate that the *p*-value is not significantly different (*p* < 0.05). Error bars represent the standard deviation.

ALP activity in cells cultured on the MBN/GO composites at both concentrations was increased compared with MBNs alone (Figure 6). On day 7, the ALP activity in cells grown on the MBN/GO composite was slightly decreased. However, on day 14, the ALP activity was significantly higher than that on day 7, and the ALP activity in cells grown on the MBN/GO composite was significantly increased compared with that in cells cultured on MBNs alone and the control. MTT and the ALP activity assays showed that the response of the hDPSCs in the 0.5 mg/mL-coated wells was pronounced and dependent on the GO concentration. Therefore, 0.5 mg/mL-coated samples were used for subsequent experiments. 

### 3.4. qRT-PCR

**Figure 7:**
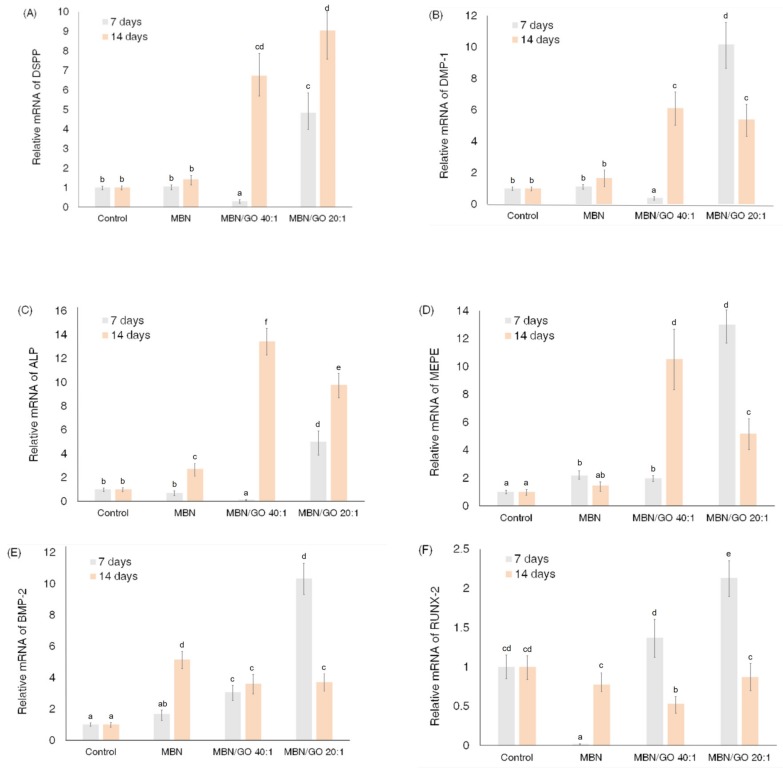
Effect of the MBN/GO composite on the expression of odontogenic differentiation markers in hDPSCs. qRT-PCR for evaluating (**A**) DSPP, (**B**) DMP-1, (**C**) ALP, (**D**) MEPE, (**E**) BMP-2, and (**F**) RUNX-2 mRNA expression in hDPSCs grown on polystyrene tissue culture plates (control), MBN, MBN/GO 40:1, and MBN/GO 20:1 for 7 and 14 days (*n* = 3).

The mRNA expression of DSPP, DMP-1, RUNX-2, BMP-2, ALP, and MEPE was investigated to evaluate the effect of the MBN/GO composite on the odontogenetic ability of hDPSCs (Figure 7). The levels of the odontogenic-specific markers, DSPP and DMP-1, in cells grown on the MBN/GO 40:1 composite were decreased compared with that in cells grown on the blank control and the MBN composite at day 7; however, these levels were significantly upregulated at day 14. The cells cultured on the MBN/GO 20:1 composite exhibited higher mRNA expression than the blank control and MBNs alone treated cells on day 7 and 14. The mRNA levels of ALP and MEPE exhibited a similar trend. The mRNA levels of RUNX-2 and BMP-2 were only affected in the MBN/GO composites after day 7. The mRNA expression of BMP-2 (in cells grown on 40:1 as well as on 20:1 composites) and RUNX-2 (in cells grown on 20:1 composites) was upregulated compared with that observed in cells grown on the blank control and on MBNs alone. ALP, MEPE, BMP-2, and RUNX-2 are mutual osteo/odontogenic markers, suggesting that the MBN/GO composites induced the odontogenesis of hDPSCs in a manner similar to osteogenesis.

### 3.5. Western Blot 

**Figure 8:**
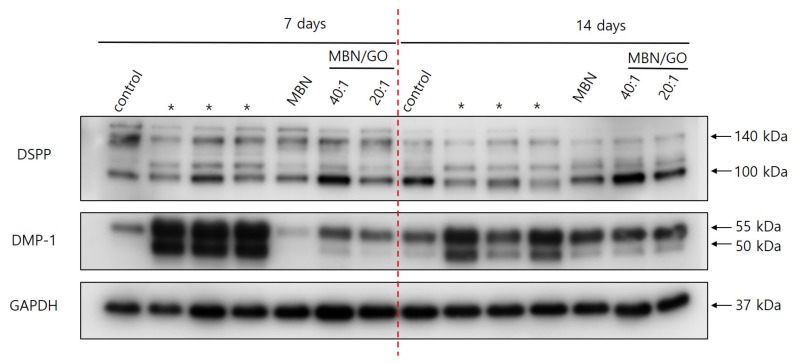
DSPP and DMP-1 expression was assessed by Western blot using glyceraldehyde 3-phosphate dehydrogenase (GAPDH) as the loading control. Lane 1: control; lane 2: cells cultured on MBNs alone; lanes 3 and 4: cells cultured on MBN/GO 40:1 and 20:1, respectively for 7 days; lanes 5–8: cells cultured on MBN/GO 40:1 and 20:1, respectively for 14 days. Asterix (*) was not included in this experiment.

Western blot was used to investigate the expression of DSPP and DMP-1, two odontogenic-specific marker proteins, in hDPSCs (Figure 8). Three separate bands for DSPP were observed. The band with the highest molecular weight (150 kDa) corresponded to the full-length DSPP, and the two ~100 kDa bands corresponded to dentine sialoprotein (DSP) and dentine phosphoprotein (DPP), the cleavage products of DSPP, respectively. The intensity of the DSPP, DSP, and DPP bands in cells grown on MBN/GO 40:1 and 20:1 composites was higher than that observed in cells cultured on MBNs alone, indicating that GO promotes DSPP expression and cleavage. In addition, the band corresponding to that of full-length DSPP was less intense on day 14 compared with that on day 7. Increased proteolytic cleavage of DSPP was observed over time, as evidenced by the increased band intensity of the cleavage products. The degradation of DMP-1 into two fragments is important for the mineralization of hDPSCs, and we were able to detect a band with a molecular weight of 57 kDa, which corresponds to the C-terminal fragment. The band corresponding to the C-terminal fragment of DMP-1 was more intense in cells grown on the MBN/GO 40:1 and 20:1 composites than that in cells grown on MBNs alone.

### 3.6. Alizarin red S Staining and Analysis 

**Figure 9:**
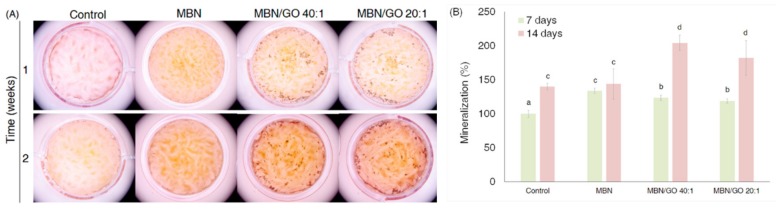
Effect of the MBN/GO composite on the mineralization of hDPSCs at 7 and 14 days. (**A**) Images of Alizarin red S-stained cells with mineralization. (**B**) Quantification of Alizarin red S staining of hDPSC cultures. ANOVA was performed to evaluate the statistical significance. The same letters indicate that the *p*-value is not significantly different (*p* < 0.05). Error bars represent the standard deviation.

Alizarin red S staining was performed to evaluate the effect of the MBN/GO composites on the mineralization ability of hDPSCs (Figure 9). Quantification of the staining showed a statistically significant increase in staining intensity compared with that in the blank control and MBNs alone for both MBN/GO 40:1 and 20:1 on day 14. Although the staining intensity was more decreased in cells grown on the MBN/GO composite than that in cells grown on MBNs alone on day 7, a significant increase in staining intensity was observed in cells grown on the MBN/GO composite on day 14. These findings indicate that the ability of the MBN/GO composite to induce mineralization is enhanced by the GO component.

### 3.7. Wnt/β-Catenin Signaling Pathway-Related Gene Expression in hDPSCs Cultured on MBN/GO Composites

**Figure 10:**
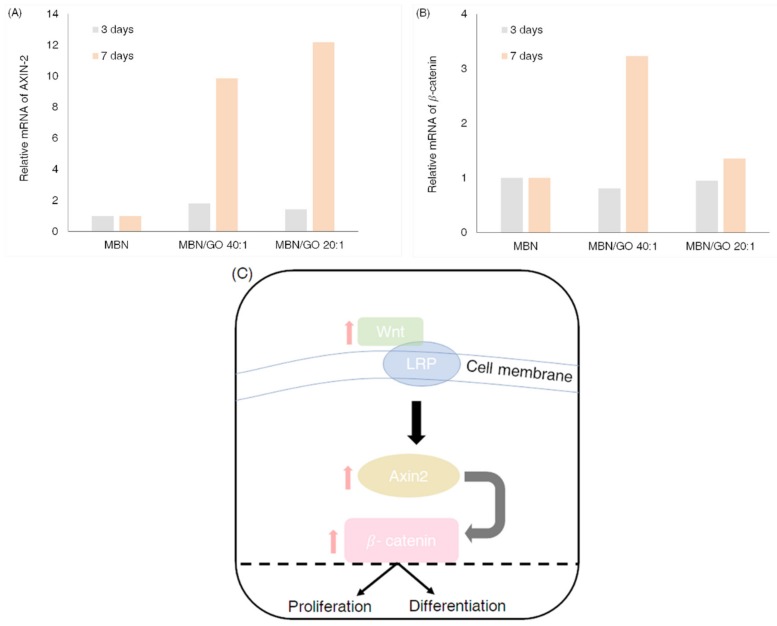
Expression of Wnt-related genes. qRT-PCR analysis for (**A**) AXIN-2 and (**B**) β-catenin in hDPSCs grown on MBN, MBN/GO 40:1, and MBN/GO 20:1 for 3 and 7 days. (**C**) The schematic diagram representing the Wnt/β-catenin signaling pathway.

qRT-PCR revealed that the expression of the Wnt-related genes, AXIN-2 and β-catenin, was upregulated in cells grown on MBN/GO composite on day 7, compared with that in cells grown on MBNs alone (Figure 10). This indicates that the MBN/GO promotes odontogenic differentiation of hDPSCs by activating the Wnt/β-catenin signaling pathway.

## 4. Discussion

The biological and mechanical properties of nanoscale bioactive glass create an advantageous environment for cell attachment. Furthermore, the combination of bioactive glass and other biomaterials creates a larger surface area/mass ratio than that obtained using conventional bioactive glass alone [21]. In addition, the mesoporous features of bioactive glass allow it to load other biomolecules and increase its bioactivity [22]. In recent years, MBNs have gained widespread use [23]. 

However, bioactive ceramics, such as MBNs, have limited applications as scaffolds in tissue engineering when used alone, due to their weak mechanical properties characterized by high brittleness and low tensile strength [7]. Therefore, several researchers have recently attempted to improve the mechanical strength of MBNs by preparing composites with various materials including GO [24]. 

Previous studies have already demonstrated improvements in mechanical properties after synthesizing composites of BAG with graphene or GO [19,25]. Porwal et al. [26] synthesized composites of 45S5 Bioglass® and graphene nanoplatelets using colloidal processing and spark plasma sintering. This composite showed enhanced mechanical strength and electrical conductivity compared with 45S5 Bioglass® alone. Fan et al. [19] and Ilyas et al. [25] successfully synthesized GO-reinforced BAG composites in a similar study, reporting a significant enhancement in the cytocompatibility and mechanical properties. 

Furthermore, GO substrates not only serve as effective reinforcements, but also act as bioactivators that promote stem cell adhesion, proliferation, and differentiation [27]. Lee et al. [11] reported an increase in the induction of osteogenesis in human mesenchymal stem cells grown on GO conjugated to bioceramics, such as calcium phosphate and hydroxyapatite. 

Bone and dentin consist of varying ratios of mineralized hydroxyapatite and collagen. The proteins expressed in both materials are also similar and include ALP and bone sialoprotein. Considering the similarities between the developmental processes of bone and dentin, we hypothesized that MBN/GO composites could induce similar responses for the odontogenic differentiation and mineralization of hDPSCs. In this study, MBN was synthesized using the sol-gel method before compositing with GO (weight ratio 40:1, 20:1) using modified colloidal processing to enhance bioactivity and strengthen the mechanical properties. The two MBN/GO composites with different weight ratios of 40:1 and 20:1 were used for in vitro tests, and their effect on hDPSCs was evaluated in vitro. 

Figure 2 shows the FESEM image of the morphology of the MBN/GO composite synthesized using modified colloidal processing. This figure shows how the spherical MBN was coated on the GO sheet. XRD, FTIR, and Raman spectroscopic investigations were subsequently performed to confirm the formation and chemical composition of the composite.

Figure 3 shows the XRD patterns of the MBN and MBN/GO composites. MBN exhibited broad bands between approximately 20 and 30° and did not have any sharp diffraction peaks, indicating that it had an amorphous structure due to the presence of silica groups [12]. Diffraction peaks were observed after the addition of GO, which can be attributed to the calcite phase (CaCO_3_, JCPDS #05-0586) (2θ = 23.0, 29.3, 35.9, 39.3, and 43.1°) [13]. The calcite phase is formed during the interaction between calcium oxide and GO. Furthermore, calcite formation has been reported when bioactive glass is combined with a carbonaceous material [28]. In a similar study, Raja et al. [12] reported that nanohybrids of bioactive glass nanorods and reduced graphene oxide exhibited a calcite mineral phase (CaCO_3_) in XRD analysis. Calcite is often used as a composite component in biomedical applications due to its high biocompatibility [14,15,16]. As such, we believe that calcite played a role in reducing the cytotoxicity of the MBN/GO composite synthesized in this study.

The FTIR spectra of the MBN, GO, and MBN/GO composites are shown in Figure 4. In the FTIR spectra of MBNs, the peaks at 472 and 795 cm^−1^ were attributed to the rocking vibration and symmetrical stretching of the Si–O–Si band, respectively [17]. Furthermore, a very strong absorption peak was detected at 1078 cm^−1^, which was attributed to Si–O–Si asymmetrical stretching [17]. A peak at 1638 cm^−1^ was associated with the adsorption of water molecules by Si–O–H [29]. For both MBN and GO, mutual broad bands were detected at approximately 3430 cm^−1^, which were attributed to the O–H stretching vibration of the adsorbed water molecules [30]. For GO, peaks were observed at 1058, 1438, 1627, and 1721 cm^−1^, corresponding to C–O stretching vibration, O–H deformation vibration, sp^2^ hybridized C=C stretching vibration, and conjugated carbonyl group (C=O) stretching vibration of the COOH group, respectively [19,31]. After the incorporation of MBNs, the band at 1721 cm^−1^, corresponding to the conjugated carbonyl group of GO, disappeared and was replaced by a new peak at around 1080 cm^−1^, corresponding to the Si–O–Si asymmetrical stretching vibration and representing the Si–OC asymmetrical stretching vibration. This indicates that the carbonyl groups of GO were converted into Si–OC bands [18]. In addition, the fact that the characteristic peaks of GO—found at 1058 and 1627 cm^−1^—disappeared suggests that some of the GO was subject to low-temperature thermal reduction during the 24-h period of drying in the vacuum oven at 65 °C (after stirring the mixed colloidal suspension of MBN and GO) [19,20]. 

The peak characteristics of GO appeared near 1356 cm^−1^ for the D band, which is associated with the disorder in edge or aromatic effect of graphene, and 1602 cm^−1^ for the G band, which is associated with the in-plane stretching vibration of the sp^2^ CC bond (Figure 5) [23,32]. The characteristic peaks of GO did not show a shift in the MBN/GO composites. As shown in Figure 2, the results obtained by Raman spectroscopy, along with XRD and FTIR, confirmed the successful incorporation of MBNs on GO to form the desired composites.

Although GO promotes a favorable environment for cell adhesion and proliferation, it can be cytotoxic at certain concentrations. Many studies have found that GO is highly biocompatible, regardless of the size and dose [33], while others have reported that it has a dose-dependent toxicity [34]. As such, the assessment of the cellular toxicity of GO is controversial. In this study, MBN/GO composites were used as scaffolds to investigate the viability of hDPSCs by forming uniform adherent layers on polystyrene tissue culture plates at two concentrations: 0.01 and 0.05% (w/v) (Figure 6). Our results showed that MBN/GO did not inhibit growth compared with MBNs alone. At both concentrations, a significant increase in cell viability was observed in the MBN/GO composite than that observed with MBNs alone, except for the MBN/GO 40:1 composite (0.1 mg/mL) after 24 h. This demonstrates that the concentration of the sample used in this study is highly biocompatible and does not result in cytotoxicity, despite direct contact with the hDPSCs. This is consistent with the results of Ilyas et al. [25], who reported higher stem cell growth in bioactive glass functionalized graphene oxide substrates compared with that observed using bioactive glass at 24 h. 

ALP activity was measured to evaluate the differentiation potential of hDPSCs in response to growth on MBN/GO composites (Figure 7). ALP is an important marker of bone- and dentin-forming cells and is used to evaluate the osteo/odontoblastic differentiation-inducing effects of various bioactive molecules [35,36]. Although the ALP activity of the hDPSCs grown on MBN/GO composites was initially inhibited compared with the control at day 7, there was a significant increase in activity, with respect to MBNs alone, after 14 days of culture. As the GO content increased in the composites, a proportional increase in ALP activity was observed. This trend was particularly prominent in the 0.5 mg/mL sample, where the increase in ALP activity was more pronounced in cells grown on the MBN/GO composite than in cells grown on the blank control or MBNs alone. This increase in ALP activity is indicative of the differentiation potential of hDPSCs. Previous studies on mesenchymal stem cells have reported similar effects in response to growth on GO components [37]. 

To further investigate the influence of MBN/GO on the odontogenesis of hDPSCs, mRNA expression of DSPP, DMP-1, RUNX-2, BMP-2, ALP, and MEPE was investigated (Figur 8). RUNX-2 is a transcription factor that regulates the expression of genes required for odontoblastic differentiation and is known to be a master regulator of odontogenesis [38]. BMP-2 and MEPE are non-collagenous proteins in the dentine matrix that play an important role in regulating odontogenic differentiation and mineralization during dentinogenesis [39]. While RUNX-2, BMP-2, ALP, and MEPE are osteogenic markers, DMP-1 and DSPP are markers specific for odontogenic differentiation. DMP-1 and DSPP are non-collagenous extracellular matrix (ECM) proteins expressed by odontoblasts, whose expression is upregulated during active mineralization [40]. qRT-PCR demonstrated that in hDPSCs cultured on MBN/GO composites, the mRNA levels of odontogenesis-related genes were upregulated compared with that in cells cultured on MBNs alone. The mRNA levels of RUNX-2 and BMP-2 increased in response to growth on MBN/GO 40:1 as well as MBN/GO 20:1 composite on day 7, compared with growth on MBNs alone. However, the mRNA levels of DMP-1, DSPP, ALP, and MEPE increased in cells cultured on the MBN/GO 20:1 composite only. Despite this, GO did not appear to have any effect on the mRNA levels of BMP-2 and RUNX-2 on day 14, whereas the mRNA levels of DMP-1, DSPP, ALP, and MEPE were significantly increased in cells grown on MBN/GO 40:1 and 20:1 composites. These results indicate that GO promoted odontogenic differentiation in hDPSCs in a manner similar to that observed in the case of osteoblastic differentiation. As reported in previous studies, RUNX-2 is a master transcription factor for skeletal and tooth development that acts at very early stages of odontogenic differentiation [41] and is downregulated as differentiation progresses. mRNA expression of DMP-1, MEPE, and BMP-2 was more downregulated on day 14 compared with that on day 7, presumably due to the fact that cell density affected the differentiation.

In this study, the expression of DSPP and DMP-1, the odontogenic-specific marker proteins of hDPSCs, was determined by Western blot analysis (Figure 9). DSPP and DMP-1 are both members of the small integrin-binding ligand N-linked glycoproteins family and play an important role in dentin mineralization and maturation [42]. As dentinogenesis progresses, DSPP is proteolytically degraded into two subunits, DSP and DPP [43]. This proteolytic processing of DSPP is referred to as the activating stage of DSPP expression [40,44]. We detected three separate bands in the Western blot for DSPP and confirmed the molecular weight of three proteins. The band with the highest molecular weight (150 kDa) represented the full-length DSPP, while the other two bands represented DSP and DPP, the cleavage products of DSPP, respectively, consistent with previous studies [45]. DSP is associated with the initiation of mineralization, and DPP is known to play an important role in the formation and growth of apatite during mineralization [46,47]. In our results, the intensity of the DSPP band was lower at day 14 compared with that on day 7, whereas the opposite was observed for the DSP and DPP bands. In particular, DPP formation was more enhanced in cells cultured on the MBN/GO composite compared with those grown on MBNs alone. These findings indicate that the degradation of DSPP progressed over time and that GO promotes the proteolytic processing of DSPP. During odontoblast maturation, DMP-1 phosphorylates and exports extracellularly to promote the mineralization of the ECM [48]. Previous studies have shown that the full-length form of DMP-1 inhibits mineralization; however, DMP-1 is degraded into two fragments, an N-terminal fragment (molecular weight, 37 kDa) and a C-terminal fragment (molecular weight, 57 kDa) [49]. Mineralization is initiated after dephosphorylation [50]. We detected a band with a molecular weight of 57 kDa, corresponding to the C-terminal fragment, indicating that the hDPSCs were primed for mineralization. The degradation of DMP-1 was promoted by the GO component, as the C-terminal fragment was present in higher abundance in cells grown on the MBN/GO composite than in cells grown on MBNs alone. This trend was clearly observed on day 7.

The results of qRT-PCR and Western blotting support the hypothesis that MBN/GO composites can induce the odontogenic differentiation of hDPSCs to promote mineralization during dentin regeneration. When hDPSCs differentiate into odontoblasts, they secrete ECM for dentin formation and initiate mineralization [51]. In this study, we analyzed calcium deposition using Alizarin red staining and verified the formation of mineralized nodules (Figure 10). Mineralization refers to the cell-mediated deposition of extracellular calcium and phosphates [52]. It has been reported that bioactive glass promotes the mineralization of stem cells, as calcium and phosphate ions become a source of calcification [53]. Similarly, the present study demonstrated increased induction of mineralization by MBNs compared to the blank control. MBN/GO composites enhanced the formation of mineralized nodules more than MBNs alone. While the mineralization of hDPSCs was inhibited in cells grown on the MBN/GO composite compared with those grown on MBNs alone on day 7, the staining intensity of hDPSCs cultured on MBN/GO composites was significantly higher than that of those cultured on MBNs alone. This suggests that the GO component of the MBN/GO composite plays an important role in the induction of mineralization in hDPSCs.

The Wnt/β-catenin signaling pathway is known to play an important role in the regulation of proliferation, differentiation, and morphogenesis of several types of stem cells [54]. In this study, the effect of MBN/GO composites on the Wnt/β-catenin signaling pathway was investigated in order to identify the regulatory mechanisms by which GO promotes the odontogenic differentiation of hDPSCs. qRT-PCR showed that the levels of AXIN-2 and β-catenin mRNA, two Wnt-related genes, were increased in the hDPSCs cultured on MBN/GO composites, compared with those cultured on MBNs alone (Figure 10). AXIN-2 is the most accurate reporter of the Wnt canonical pathway, as it directly receives the signal transduced by the Wnt ligand and regulates the stability of β-catenin [55]. β-catenin, also known as Cadherin-Associated Protein Beta (CTNNB), is an important gene in the Wnt/β-catenin signaling pathway that regulates phosphorylation by AXIN-2 signaling, and translocates into the nucleus to bind to specific transcription factors when its intracellular concentrations increase [56]. In their study evaluating the osteogenic capacity of human bone marrow stromal cells in response to GO-modified β-tricalcium phosphate, Wu et al. [57] argued that GO regulates osteogenic induction via the activation of the Wnt/β-catenin signaling pathway. To the best of our knowledge, the present study is the first to report the association between the odontogenic differentiation capacity of GO and Wnt-related signaling pathways in the field of dentin-pulp complex tissue engineering. In this study, both MBN/GO 40:1 and 20:1 composites were found to upregulate the expression of AXIN-2 and β-catenin in hDPSCs compared with that in hDPSCs cultured on MBNs alone at day 7. These results demonstrate that MBN/GO enhances odontogenic differentiation by activating the Wnt/β-catenin signaling pathway in hDPSCs.

## 5. Conclusions

Our results show that MBN/GO composites enhance the mRNA and protein expression of odontogenic differentiation markers in hDPSCs, and promote mineralization, an essential process for dentin regeneration. The MBN/GO composites enhanced odontogenic differentiation via the Wnt/β-catenin signaling pathway. The results of the present study suggest that MBN/GO composites may promote the differentiation of hDPSCs into odontoblast-like cells and may therefore be used to induce dentin formation in dentin-pulp complex tissue engineering.

## Figures and Tables

**Table 1:** Quantities of mesoporous bioactive glass nanoparticle (MBN) and MBN/graphene oxide (GO) powder coated on culture plates.

Sample Powder’s Concentration % (w/v)	Samples	
	0.05%	0.01%
Surface coating concentration at culture plates (mg·cm^–2^)	0.15625 mg·cm^–2^	0.03125 mg·cm^–2^

**Table 2:** Primer sequences for quantitative real-time polymerase chain reaction.

Gene	Primer	Sequence (5’-3’)	Size (bp)
**β-actin**	Forward	GCACTCTTCCAGCCTTCCTT	150
	Reverse	AATGCCAGGGTACATGGTGG	
**DMP-1**	Forward	GGAGAGACAGCAAGGGTGAC	87
	Reverse	CACTGCTGGGACCATCTACG	
**DSPP**	Forward	GCTGGCCTGGATAATTCCGA	135
	Reverse	CTCCTGGCCCTTGCTGTTAT	
**ALP**	Forward	AATGTGGACACAGTGGCTGGA	78
	Reverse	TCTCCTGCTCAGTCATCTGCT	
**BMP-2**	Forward	AAGCCAAACACAAACAGCGG	104
	Reverse	GGGAGCCACAATCCAGTCAT	
**RUNX-2**	Forward	TCTGGCCTTCCACTCTCAGTA	134
	Reverse	TGGATAGTGCATTCGTGGGT	
**MEPE**	Forward	GCAGCTATCCACACCAGAAAG	113
	Reverse	GTTGAAATGTTGGTGCTGCC	
**AXIN-2**	Forward	CCCTGCTGACTTGAGAGAGAC	82
	Reverse	CCCACTGAGTCTGGAATCTC	
**β-catenin**	Forward	CAGCGTGGACAATGGCTACT	101
	Reverse	AGATTCCTGCTGGTGGCTTG	

Abbreviations: DMP-1, dentine matrix protein 1; DSPP, dentin sialophosphoprotein; ALP, alkaline phosphatase; BMP-2, bone morphogenetic protein 2; RUNX-2, runt-related transcription factor 2; MEPE, matrix extracellular phosphoglycoprotein; AXIN-2, axis inhibition protein-2.
